# Intestinal Microbiota Increases Cell Proliferation of Colonic Mucosa in Human-Flora-Associated (HFA) Mice

**DOI:** 10.3390/ijms25116182

**Published:** 2024-06-04

**Authors:** Giovanni Brandi, Carlo Calabrese, Simona Tavolari, Chantal Bridonneau, Pierre Raibaud, Giuseppina Liguori, Muriel Thomas, Monica Di Battista, Valerie Gaboriau-Routhiau, Philippe Langella

**Affiliations:** 1 Department of Medical and Surgical Science, University of Bologna, 40138 Bologna, Italy; carlo.calabrese2@unibo.it (C.C.); liguori.gy@gmail.com (G.L.); monica.dibattista@unibo.it (M.D.B.); 2 Medical Oncology, IRCCS Azienda Ospedaliero-Universitaria di Bologna, 40138 Bologna, Italy; simona.tavolari@aosp.bo.it; 3 INRAe, AgroParisTech, Micalis Institute, Université Paris-Saclay, 78350 Jouy-en-Josas, France; chantal.bridonneau@orange.fr (C.B.); pierre.raibaud@jouy.inra.fr (P.R.); muriel.thomas@jouy.inra.fr (M.T.); valerie.gaboriau-routhiau@inserm.fr (V.G.-R.); philippe.langella@jouy.inra.fr (P.L.); 4 Laboratory of Intestinal Immunity, Imagine Institute, INSERM UMR1163, Université Paris Cité, 75015 Paris, France

**Keywords:** intestinal microbiota, cell proliferation, germ-free mice, holoxenic mice, HFA mice

## Abstract

Intestinal epithelium renewal strictly depends on fine regulation between cell proliferation, differentiation, and apoptosis. While murine intestinal microbiota has been shown to modify some epithelial cell kinetics parameters, less is known about the role of the human intestinal microbiota. Here, we investigated the rate of intestinal cell proliferation in C3H/HeN germ-free mice associated with human flora (HFA, n = 8), and in germ-free (n = 15) and holoxenic mice (n = 16). One hour before sacrifice, all mice were intraperitoneally inoculated with 5-bromodeoxyuridine (BrdU), and the number of BrdU-positive cells/total cells (labelling index, LI), both in the jejunum and the colon, was evaluated by immunohistochemistry. Samples were also observed by scanning electron microscopy (SEM). Moreover, the microbiota composition in the large bowel of the HFA mice was compared to that of of human donor’s fecal sample. No differences in LI were found in the small bowels of the HFA, holoxenic, and germ-free mice. Conversely, the LI in the large bowel of the HFA mice was significantly higher than that in the germ-free and holoxenic counterparts (*p* = 0.017 and *p* = 0.048, respectively). In the holoxenic and HFA mice, the SEM analysis disclosed different types of bacteria in close contact with the intestinal epithelium. Finally, the colonic microbiota composition of the HFA mice widely overlapped with that of the human donor in terms of dominant populations, although *Bifidobacteria* and *Lactobacilli* disappeared. Despite the small sample size analyzed in this study, these preliminary findings suggest that human intestinal microbiota may promote a high proliferation rate of colonic mucosa. In light of the well-known role of uncontrolled proliferation in colorectal carcinogenesis, these results may deserve further investigation in a larger population study.

## 1. Introduction

Intestinal epithelium renewal strictly depends on fine regulation between cell proliferation, differentiation, and apoptosis [1]. Although the intimate molecular mechanisms driving these processes have largely been identified, the impacts of the two main endoluminal factors, namely dietary components and microorganisms, still remain to be fully elucidated.

A high intake of processed foods rich in refined sugars, salt, animal proteins, and trans/saturated fats, as occurs in a Western diet, has been associated with intestinal proliferative patterns and with an increase in colorectal cancer (CRC) risk, mainly due to the induction of chronic inflammation and tissue acidosis, which can promote malignant cell transformation [2,3,4].

Conversely, less is known about the role of the intestinal microbiota. The adult human digestive apparatus harbors up to 10^14^ bacteria, which may colonize (autochthonous bacteria) or transiently pass through (allochthonous bacteria) the gastrointestinal tract [5]. More than 90% of the dominant bacteria of the autochthonous intestinal population belong to four phyla: *Proteobacteria*, *Actinobacteria*, *Firmicutes*, and *Bacteroidetes*, with six genera belonging to strict anaerobes (*Bacteroides*, *Eubacteria*, *Bifidobacteria*, *Clostridia*, *Peptostreptococci*, and *Ruminococci*) structured around three specific enterotypes based on *Bacteroides* (Enterotype 1), *Prevotella* (Enterotype 2), and *Ruminococcus* (Enterotype 3), respectively [5,6]. The high redundancy of the gut bacterial species is reflected in the richness and diversity of the microbial community and their genomes, which ensure diverse functionality and overall stability. The microbiota and their related genome, namely microbiome, form a complex ecological community that profoundly impacts intestinal homeostasis and disease states. Microbiota/microbiome organization is far from to be random, and each individual has their own specific composition that remains quite stable during adult life. Diet changes, pathologies, and specific drugs (i.e., antibiotics or chemotherapy treatments) may modify this balance, carrying over a new point of stability [7,8,9].

The relationship between the host cells and microbial populations of the human gastrointestinal tract constitutes a complex ecosystem. Indeed, the microbiota convey a complex pool of prokaryote genes, able to extract energy from food and involved in the catabolism of several elements derived from the diet. The microbiota also regulates the immune response (both innate and adaptive), protects against pathogens, interferes with drug metabolism, modifies the characteristics of the intestinal epithelium, and is involved in redox stress cell damages, cell motility, angiogenesis, proliferation, and differentiation [9,10].

A link between the microbiota composition and the development of intestinal diseases, including CRC, has emerged in recent years [11]. Metagenomic studies have demonstrated that, compared to the gut microbiota of healthy individuals, the gut microbiota become dysregulated in CRC patients, with a higher species richness, lower abundance of potentially protective taxa, and increased abundance of pro-carcinogenic ones (such as *Bacteroides*, *Escherichia*, and *Fusobacterium*) [12]. Microbiota alterations have also been observed in adenomas, the early stage of CRC carcinogenesis [11]. The underlying mechanisms linking the microbiota to CRC carcinogenesis are complex and not yet fully elucidated; however, the release of pro-inflammatory mediators, microbial-derived factors such as metabolites or genotoxins, host immunological dysfunction, and an imbalance in nutrient absorption and energy metabolism have been established as promoting factors [13].

In recent years, the availability of germ-free and human-flora-associated (HFA) animal models has fostered studies on the main morpho-functional changes induced by the human microbiota along the digestive tract and elsewhere [14]; however, the role of microbiota in intestinal proliferation still remains to be clarified.

Murine microbiota has been reported to modify some cell kinetics parameters of the intestinal epithelium in previous studies. An increased proliferation rate of the duodenal epithelium was indeed observed in conventional mice in comparison to germ-free ones [15]; similarly, a higher capacity of cycling was shown in the colonic epithelial cells of conventional mice compared to germ-free mice [16]. Interestingly, in our previous work, we reported the induction of up to 27% of the genes involved in cell cycle regulation in HFA mice 60 days post-colonization, compared to only 3% of induced genes in holoxenic mice [17]. This finding prompted us to hypothesize that the human microbiota could affect intestinal cell proliferation. As hyperproliferation represents an early and constant phenotypic trait in gastrointestinal cancer development (including CRC) and can be considered as a marker of cancer risk [18], the present study aimed to investigate the effect of human intestinal microbiota on the growth rate of the intestinal epithelium in HFA mice models.

## 2. Results

### 2.1. Intestinal Cell Proliferation in Germ-Free, Holoxenic, and HFA Mice

In order to investigate the role of human microbiota in regulating intestinal cell proliferation, we compared 5-bromodeoxyuridine (BrdU) incorporation in the jejunum and colon tissues from 15 germ-free, 16 holoxenic, and 8 HFA mice. As shown in Table 1, a mean number ranging from 63 to 76 crypts was analyzed for each single sector of the jejunum and colon. The number of cells per crypt ranged from 25 to 32, with the total number of analyzed cells ranging from 1861 to 2010; BrdU^+^ cells ranged from 155 to 185 in the jejunum of the mice, whereas from 59 to 109 in the colon (Table 1).

**Figure 1:**
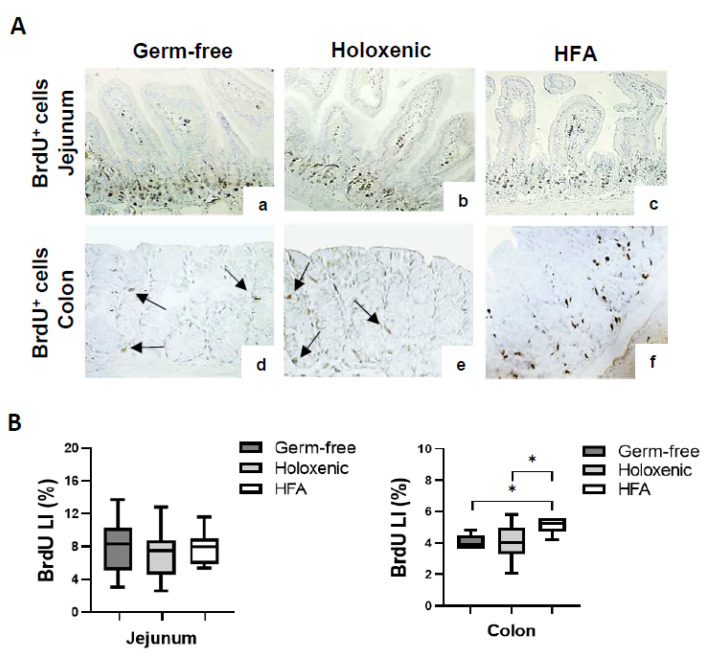
(**A**) Immunohistochemical analysis of BrdU^+^ cells in the jejunum (**a**–**c**) and colon (**d**–**f**) tissue from germ-free, holoxenic, and HFA mice. BrdU labels the nuclei of proliferating cells in the S phase of the cell cycle, and labelled cell nuclei appear brown. Black arrows (**d**,**e**) show some BrdU^+^ cells in the colon of germ-free and holoxenic mice. Magnification 10×. (**B**) Box plots of BrdU LI values, LI median (bold line in the box), and interquartile range (upper and lower lines of the box) in jejunum and colon of germ-free, holoxenic, and HFA mice. * *p* < 0.05.

An immunohistochemical analysis of BrdU^+^ cells revealed a median BrdU LI of 8.36% (±2.82), 7.71% (±3.23), and 8.29 (±2.07) in the jejunum of the germ-free, holoxenic, and HFA mice, respectively, with no statistical difference observed among these subgroups (Figure 1A,B). In the colon, the number of BrdU^+^ cells was lower than that in the jejunum, with a median BrdU LI reaching 3.94% (±1.32), 4.29% (±1.11), and 5.94% (±1.26) in the germ-free, holoxenic, and HFA mice, respectively (Figure 1A,B). Notably, the number of BrdU^+^ cells was significantly higher in the colon tissue from the HFA mice compared to the germ-free and holoxenic mice (*p* = 0.017 and *p* = 0.048, respectively). Overall, these findings suggest that human intestinal microbiota can increase the proliferation rate of colonic epithelial cells in HFA mice.

### 2.2. Bacteria Distribution along the Intestine of Germ-Free, Holoxenic, and HFA Mice

**Figure 2:**
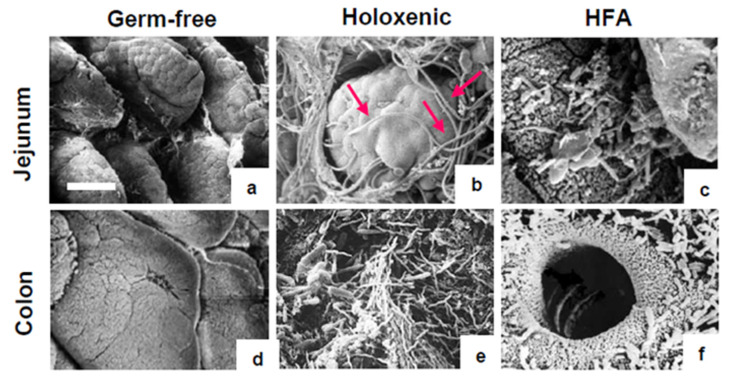
SEM analysis of jejunum and colon in germ-free, holoxenic, and HFA mice. No bacteria are detected on the intestinal mucosa of germ-free mice (**a**,**d**), whereas different types of bacteria are present in the jejunum and colon of holoxenic and HFA mice (**b**,**c**,**e**,**f**). In holoxenic mice, many segmented filamentous bacteria (SFB) are observed in the jejunum ((**b**), red arrows), while a different typology of bacteria in close contact with the epithelium is found in the colon (**e**). Several bacilli-like bacteria are found in HFA mice: in the colon, the bacteria are facing at the apex of a crypt (**f**). Bar = 10 μm.

To better understand the spatial distribution of bacteria in the jejunum and colon of the mice, a scanning electron microscopy (SEM) analysis was performed in the germ-free, holoxenic, and HFA models. As expected, no bacteria were found in the jejunum and colon mucosa of the germ-free mice (Figure 2, panels a,d); conversely, different types of bacteria were found in these intestinal segments in both the holoxenic (Figure 2, panels b,e) and HFA (Figure 2, panels c,f) mice. In particular, the jejunum of the holoxenic mice showed the presence of segmented filamentous bacteria (SFB), which have been specifically linked to education of the gut immune system in mice [17]. Although no conclusive data can bedrawn, recent studies suggest the occurrence of SFB also in humans [19]. In the colon, despite differences in the bacterial population were observed between holoxenic and HFA mice, according to the bacterial morphologies, both groups showed bacteria in close contact with the intestinal epithelium (Figure 2, panels e,f). 

### 2.3. Bacteriological Analysis of Fecal Flora from Healthy Donor and HFA Mice

**Figure 3:**
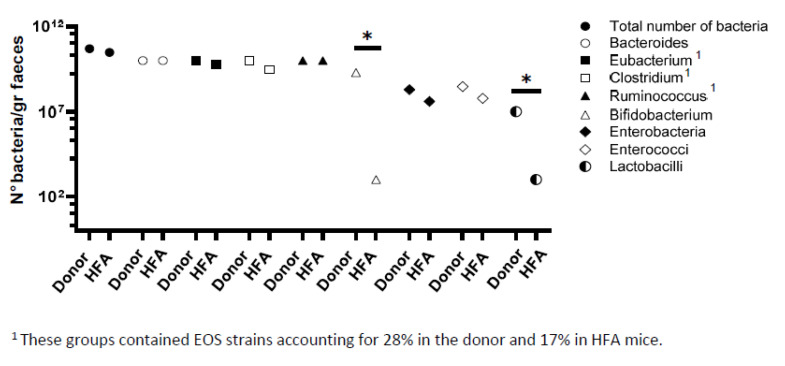
Bacteriological analysis of the fecal colon content from human donor and HFA mice. No significant differences are found in terms of total bacterial count or numbers of *Bacteroides*, *Ruminococcus*, *Eubacterium Clostridium*, *Enterobacteria*, and *Enterococci*. Conversely, *Bifidobacteria* and *Lactobacilli* significantly decreased in HFA mice in comparison to human donor. * *p* < 0.05.

We next performed a bacteriological analysis of the fecal flora from the HFA mice and the corresponding human healthy feces’ donor. For this purpose, we chose traditional cultural methods, as, compared to sequencing/metagenomics approaches, they can increase the microbial identification from the human gut up to 30% [20,21]. Indeed, although a sequencing/metagenomic analysis can provide greater information about the complex genome of the microbiota, it has the limit of missing minority bacteria populations (including those with a potential pathogenic role) and does not allow for evaluating the role of individual bacterial species in gnotobiotic animal models [21]. As reported in Figure 3, the microbiota composition was comparable between the fecal flora from the donor and HFA mice in terms of the total bacterial count and numbers of *Bacteroides*, *Ruminococcus*, *Eubacterium*, and *Clostridium*, which remained the dominant populations. *Enterobacteria* and *Enterococci* were detected at a subdominant level in both the human and HFA mice; conversely, both *Bifidobacteria* and *Lactobacilli* disappeared in the colon content of the HFA mice compared to the fecal flora from the human donor (Figure 3).

## 3. Discussion

**Figure 4:**
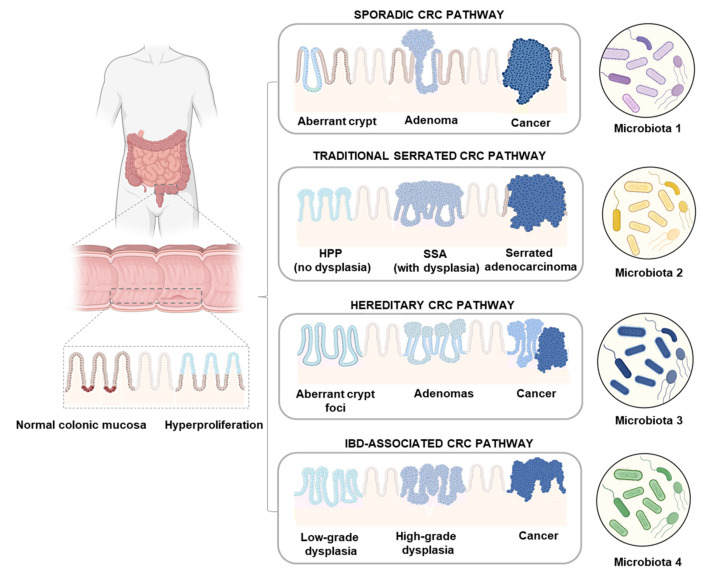
CRC development in canonical, traditional serrated, hereditary, and IBD-associated pathways. Hyperproliferation of normal colonic mucosa represents an early and constant event in CRC carcinogenesis in all these pathways.

The increase in colonic mucosa proliferation represents an early and constant phenotypic event in CRC development, occurring in different pathways of CRC carcinogenesis [18,22,23] (Figure 4).

This persistent hyperproliferative stimulus of the colonic mucosa, resulting from host genetic determinants, dietary habits, and environmental exposure, may provide a fertile ground for malignant cell transformation, promoting the accumulation of molecular alterations over time [22,24].

A role of dietetic components in sustaining intestinal cell proliferation has been reported in some studies [25,26,27]; however, less studies are currently available about the contribution of human intestinal microbiota. Pre-clinical studies have shown that germ-free mice are less prone to develope intestinal tumors than conventionally reared mice [28]; furthermore, distinct alterations in the microbiota composition have been reported across the major stages of CRC carcinogenesis [29,30], suggesting a role of specific bacterial communities in this process.

In the present study, we investigated the role of the human microbiota in promoting the early step of intestinal carcinogenesis, namely, the epithelial proliferation of the small and large bowel using HFA mice models. A similar mouse strain and human feces donor have already been used in former studies [17]. As observed in the germ-free and holoxenic mice, no significant changes in the proliferation rate of the small bowel epithelium were detected in the HFA mice. By contrast, we found that the human intestinal microbiota induced hyperproliferation in the large bowel of the HFA mice, with a significantly higher LI than that in the germ-free and holoxenic mice. These findings are in line with a study on conventionalized mice showing that the microbiota upregulate the expression of approximately 10% of a host’s genes, especially those involved in cell proliferation [31]. Notably, this effect was highly site-specific, as the transcriptional response was more prominent in the colon than in the ileum and differed between the tip and crypt fractions [31].

The increase in colonic mucosa proliferation in the HFA mice can be ascribed to different mechanisms, many of which still remain to be fully elucidated. Firstly, bacteria could mechanically increase cell exfoliation on the top of the colon crypts and/or of small bowel villi, thereby stimulating a secondary trophic reaction on the bottom of the crypts, presumably involving autocrine or hormonal phenomena [32]. Secondly, the microbiota composition has been shown to clearly differ between the small and large bowel, both in holoxenic and HFA mice [33]. Bacteria producing butyrate (representing 60–70% of enterocytes’ source of energy) could be present in different concentrations along the gastrointestinal tract, depending on the encompassing ecosystem (normal mucosa, dysplasia, or cancer) [34]. As butyrate is known to induce epithelial cellular proliferation and differentiation [35], this different bacteria distribution could account (at least in part) for the different proliferative patterns observed between the small and large bowel.

It could be pointed out that conventionalized mice (namely, ex-germ-free mice colonized at an adult age with the whole fecal mouse microbiota, as performed with human microbiota in the HFA model), rather than holoxenic ones, may represent the most proper control for HFA mice. However, it has been shown that the transitory hyperproliferative boost induced by the microbiota in conventionalized mice (usually occurring 2 days after inoculation) is compensated by increases in p21 and p27 (and a concomitant decrease in Bcl-2) protein expression, leading to the controlled homeostasis of the colonic epithelium [16]. Moreover, a recent paper reported that conventionalized mice maintain a maximum of 80% similarity with the inoculated microbiota; therefore, their microbiota composition does not fully recapitulate that of holoxenic mice [36]. On this basis, it is unlikely that a comparison between HFA and conventionalized mice would provide significantly different results from those obtained using holoxenic ones. 

Some concerns can be also raised from the use of HFA mice as an experimental model, as significant differences between murine and human microbiota exist in terms of composition, distribution along the gastrointestinal tract, and interaction with the epithelium/mucus layer. Most constituents of the human microbiota can indeed colonize rodents remaining stable over time, but some bacterial genera do not colonize the murine gut; in particular, *Bifidobacteria* and *Lactobacilli* are spontaneously eliminated [37], as also confirmed by our results. Moreover, rodents show an intimate relationship between the mucosa and a large number of bacteria (often clustered over the mucus gel or in direct contact with epithelial cells), whereas in humans, this correlation is not detected [38]. Undoubtedly, these differences call for a precautionary approach when translating results obtained in HFA mice to humans. Despite these limitations, because life in germ-free conditions is not achievable for humans, HFA mice represent the only available (and still not substitutable) model to study many aspects of the human intestinal microbiota in relation to mucosal features and diet changes [14].

Overall, the present study suggests that the human intestinal microbiota can induce a condition of hyperproliferation in the colonic mucosa of HFA mice. The fecal microbiota for the HFA mice creation was obtained from a single human donor, so these findings have to be considered as preliminary and need to be confirmed in a larger donor population. However, as the microbiota composition varies among humans, it is conceivable that its contribution to promoting colonic epithelium proliferation may significantly differ among individuals. In this scenario, if a link among the intestinal microbiota, colonic mucosa hyperproliferation, and increased CRC risk is confirmed in future studies, such a risk should be stratified depending on the peculiar microbiota composition of each individual.

## 4. Materials and Methods

### 4.1. Animals

C3H/HeN male mice (holoxenic mice), 10 to 12 weeks old, were purchased from Janvier Labs (Le Genest-Saint-Isle, France) and reared at the Commensal and Probiotics-Host Interactions Laboratory INRAe (Jouy-en-Josas, France). Germ-free mice, obtained from the germ-free rodent-breeding facilities of the MICALIS unit at INRAe, and HFA mice were kept inside flexible film isolators in standard macrolon cages (five mice/cage), with sterile wood shavings as bedding. The animals were given free access to autoclave tap water and a standard pellet diet sterilized by gamma irradiation at 45 kGy. The isolators were maintained under controlled conditions of light (07.30–19.30 h), temperature (20–22 °C), and humidity (45–55%).

The HFA mice were generated by colonizing the germ-free C3H/HeN mice with the human fecal microbiota from the same healthy male volunteer of our previous work, with no evidence of gastrointestinal or hepatic disorders and without laxative or antibiotic use for many years [17]. For this purpose, a freshly passed stool sample was provided in an anaerobic box. Within one hour after defecation, 1 g of feces was transferred to an anaerobic cabinet, diluted 100-fold in liquid LCY medium (casitone 2 g, yeast extract 2 g, NaCl 5 g, KH_2_PO_4_ 1 g, pH 7.0 per liter), and thoroughly homogenized with an Ultra Turrax. The 10^−2^ fecal dilution was then transferred into the isolator and all germ-free mice were dosed with 0.5ml of the fecal suspension through the orogastric route. A similar treatment was performed at a 24 h interval. The HFA mice were sacrificed 30 days after colonization (time for bacterial stability).

### 4.2. BrdU Injection

5-bromodeoxyuridine (BrdU) is commonly used for the detection of proliferating cells in living tissues, and is incorporated into the newly synthesized DNA of replicating cells during the S-phase of the cell cycle [14]. Accordingly, one hour before sacrifice, the germ-free, holoxenic, and HFA mice were intraperitoneally inoculated with 100 mg/kg BrdU (Sigma, St. Louis, MO, USA) in phosphate buffer 0.1 M at pH 7.2, and sacrificed by exsanguination between 10.00 h and 11.00 h to avoid any circadian interference on the proliferative pattern. For each animal, the abdomen was opened through a midline incision and the gastrointestinal tract was removed. 

### 4.3. BrdU Immunohistochemistry

Samples of jejunum and colon from the germ-free, holoxenic, and HFA mice were formalin-fixed and paraffin-embedded. Serial 3 μm sections from each specimen were stained with hematoxylin and eosin for a histological analysis or treated with 3% H_2_O_2_ for 10 min, 0.1% trypsin for 6 min, and HCl (2 mol/L) for 30 min to denature the DNA for the BrdU immunohistochemical analysis. The sections were then incubated with mouse anti-BrdU antibody (GeneTex, Inc., San Antonio, TX, USA) overnight at 4 °C, followed by incubation for 30 min at room temperature with Anti-Mouse/Rabbit IgG VisUCyte HRP Polymer Antibody (R&D Systems, Minneapolis, MI, USA). The sections were developed in 3,3′-diaminobenzidine and counterstained with hematoxylin. For each animal, the number of BrdU-positive cells/total cells (labelling index, LI) along the crypt examined was counted for both the small and large bowel. In the selected glands, the entire length of the crypt had to be visible in the section, and the base of the crypt had to be in contact with the muscularis mucosa. The total number of cells per crypt column (a single column of cells on each side of the length of the crypt) and the number of BrdU-positive cells were counted in all samples.

### 4.4. Scanning Electron Microscopy (SEM)

The samples of the jejunum and colon were fixed in 5% glutaraldehyde in 0.1 phosphate buffer (pH 7.2) for 15 to 30 min at 4 °C. After several washings in buffer, the samples were dehydrated in serial solutions of ethanol (10%, 30%, 50%, 75%, and 95%) for 15 min, each at 4 °C to 5 °C. After a passage in absolute ethanol at room temperature, the samples were further dehydrated by critical point drying Balzers 010 (Vitechparts, Geleen, The Netherlands, then placed on specific aluminum plates and covered with gold-conducting film with Balzers MED 010 (Vitechparts, Geleen, The Netherlands). The specimens were observed with a Philips SEM 515 (Eindhoven, The Netherlands) at an acceleration between 8 and 15 kV.

### 4.5. Bacteriological Analysis

The colon content of the HFA mice and feces of the human donor were weighed, placed in the anaerobic cabinet, carefully homogenized, and diluted tenfold in LCY liquid medium. The total bacterial counts in both aerobic and anaerobic conditions were obtained by plating 0.1 mL of the dilutions onto two sets of plates containing BHI-YH agar medium (BHI DIFCO Laboratories supplemented with 5 g/L of yeast extract DIFCO and 5 mg of hemin (Sigma-Aldrich Chime, Quentin Fallavier, France). One set was incubated inside and the other outside the anaerobic cabinet for the total anaerobic and aerobic counts, respectively. Incubation was for 3 days at 37 °C. The bacterial diversity of the dominant population of strictly anaerobic bacteria was assessed by picking 96 isolated colonies of each shape from the 10^−9^ and 10^−8^ dilution plates and sub-culturing them in streaks on BHI-HY medium [30]. This set of plates was prepared in duplicate and incubated for three days at 37 °C. The first subset was completely processed in the anaerobic cabinet and the other was maintained for one hour outside the cabinet and then reintroduced into the cabinet. Colonies that did not grow after this air contact were referred to as extremely oxygen sensitive (EOS). The percentage of EOS bacteria was, thus, determined by direct comparison with the series not exposed to oxygen. All the 96 strictly anaerobic colonies grown on the medium were directly examined under a Nikon phase-contrast microscope and subsequently classified into four groups. The *Bacteroides* group contained Gram-negative, catalase-negative, or weakly positive non-spore-forming rods of a similar shape and size. The *Ruminococcus* group contained Gram-positive, catalase-negative, non-spore-forming cocci in pairs or in chains of a different shape and size. The *Eubacterium* group contained Gram-positive, catalase-negative, non-spore-forming rods of a different shape and size. The *Clostridium* group contained Gram-positive or Gram-variable catalase-negative rods of a different shape and size forming terminal or sub-terminal spores, and lastly, the EOS group contained bacteria belonging to the *Ruminococcus*, *Eubacterium*, and *Clostridium* groups [39].

### 4.6. Statistical Analysis

All results are expressed as mean ± SD. The Mann–Whitney U-test was carried out to compare the data from the germ-free, holoxenic, and HFA mice using the PRISM software (Ver 5.01, GraphPad Software, San Diego, CA, USA). A *p*-value < 0.05 was considered to be statistically significant.

## Figures and Tables

**Table 1:** Cell proliferation analysis in the jejunum and colon from germ-free, holoxenic, and HFA mice (mean number ± SD).

	Germ-Free		Holoxenic		HFA	
	Jejunum	Colon	Jejunum	Colon	Jejunum	Colon
**Crypts analysed**	70 ± 3.2	76 ± 3.7	70 ± 3.5	65 ± 2.9	73 ± 3.6	63 ± 2.7
**Cells per crypt**	28 ± 0.9	25 ± 0.3	28 ± 0.6	30 ± 1.1	29 ± 0.7	32 ± 0.5
**Total n° cells**	1940 ± 142	1910 ± 130	1955 ± 196	1861 ± 135	2109 ± 191	2010 ± 130
**BrdU^+^ cells**	165 ± 66.4	58.8 ± 26.3	155 ± 75.4	68.7 ± 20.1	184 ± 58.9	109 ± 31.5
**Labelling index (%)**	8.36 ± 2.82	3.74 ± 1.32	7.71 ± 3.23	4.29 ± 1.11	8.29 ± 2.07	5.94 ± 1.26

## Data Availability

All data generated or analyzed during this study are included in this article. Further enquiries can be directed to the corresponding author.
